# Crystal structure and Hirshfeld surface analysis of (*Z*)-*N*′-(4-meth­oxy­benzo[*d*]thia­zol-2-yl)-3,6-di-*p*-tolyl-1,2,4-triazine-5-carboximidamide

**DOI:** 10.1107/S2056989026007760

**Published:** 2026-08-04

**Authors:** Yaroslav K. Shtaitz, Dmitry S. Kopchuk, Vasiliy S. Gaviko, Nizami A. Ekberov, Mehmet Akkurt, Gizachew Mulugeta Manahelohe, Khudayar I. Hasanov

**Affiliations:** a The Ural Federal University named after the first president of Russia B N Yeltsin Mira 19 st 620002 Yekaterinburg Russian Federation; b Postovsky Institute of Organic Synthesis, Ural Branch of the Russian Academy of Sciences, Sof’i Kovalevskoy 22 st., 620137, Yekaterinburg, Russian Federation; c Sirius University of Science and Technology, Olympic Avenue 1, 354340, Sirius Federal Territory, Russian Federation; d M. N. Mikheev Institute of Metal Physics of the Ural Branch of the Russian Academy of Sciences, Sof’i Kovalevskoy 18 st., 620108, Yekaterinburg, Russian Federation; e Azerbaijan State Pedagogical University, 68 Uzeyir Hajibeyov St., AZ 1000, Baku, Azerbaijan; f Department of Physics, Faculty of Sciences, Erciyes University, 38039 Kayseri, Türkiye; g Department of Chemistry, University of Gondar, PO Box 196, Gondar, Ethiopia; h Azerbaijan Medical University, Scientific Research Centre (SRC), A. Kasumzade St. 14, AZ 1022, Baku, Azerbaijan; Tokyo University of Science, Japan

**Keywords:** crystal structure, 1,3-benzo­thia­zole, 1,2,4-triazine, hydrogen bonds, Hirshfeld surface analysis

## Abstract

The title mol­ecule, C_26_H_22_N_6_OS, has a stable conformation due to two intra­molecular N—H⋯·N hydrogen bonds that produce fused *S*(5) and *S*(6) rings. Layers parallel to the (010) plane are formed in the crystal by inter­molecular C—H⋯O and C—H⋯N hydrogen bonding. Additionally, C—H⋯π and π–π [centroid-to-centroid distance = 3.7380 (12) Å] inter­actions link the mol­ecules, creating ribbons along the *a*-axis direction. The inter­molecular inter­actions were qu­anti­fied using Hirshfeld surface analysis and two-dimensional fingerprint plots, which revealed the relative contributions of H⋯H (48.6%), C⋯H/H⋯C (18.8%), and N⋯H/H⋯N (12.0%) contacts to the crystal packing.

## Abstract 2

In the crystal, the mol­ecules are linked by inter­molecular C—H⋯O and C—H⋯N hydrogen bonds, forming sheets parallel to the (010) plane. Furthermore, mol­ecules are connected by C—H⋯π and π–π [centroid-to-centroid distance = 3.7380 (12) Å] inter­actions, forming ribbons along the *a*-axis direction.

## 1. Chemical context

Heterocyclic amidine derivatives of benzo­thia­zole are notable pharmacophores with anti-inflammatory (Sondhi *et al.*, 2011 ▸; Racane *et al.*, 2023 ▸) and neuroprotective (Anzini *et al.*, 2010 ▸) activities. In recent years, benzo­thia­zole-triazine hybrid mol­ecules have been reported as potent anti-tumor agents (Kumar *et al.*, 2015 ▸) and COX-2 inhibitors (Ertas *et al.*, 2022 ▸).
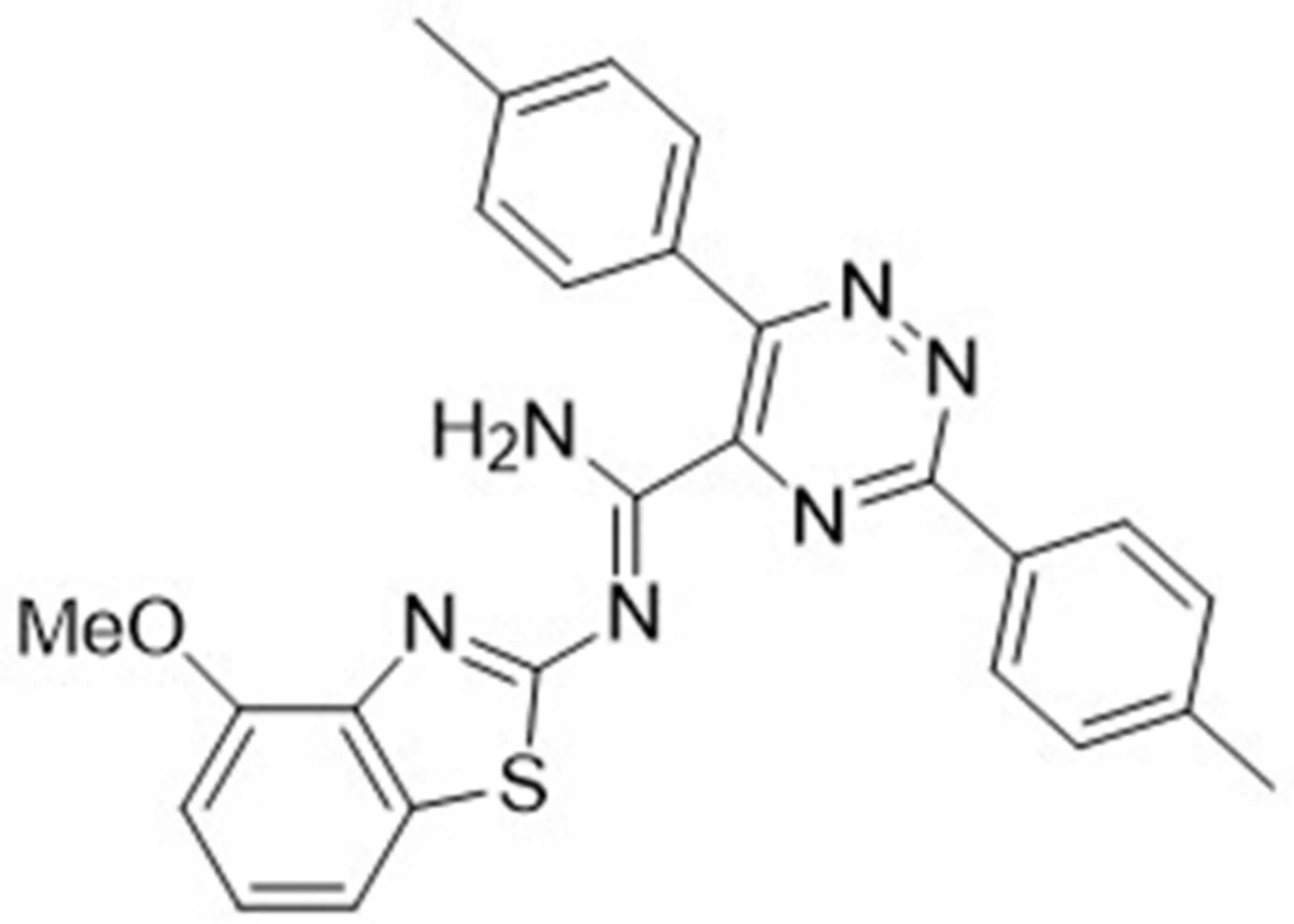


Previously, we have developed a method for modification of 5-cyano-1,2,4-triazines with heterocyclic amines *via* a nucleophilic *ipso*-substitution reaction (Krinochkin *et al.*, 2022 ▸; Rammohan *et al.*, 2021 ▸), thus as our next step we have tried to apply 2-amino­benzo­thia­zoles to this methodology. It was found that solvent-free inter­action of 3,6-di-*p*-tolyl-1,2,4-triazine-5-carbo­nitrile (**1**) with 4-meth­oxy­benzo[*d*]thia­zol-2-amine (**2**) at 423 K leads to the product (*Z*)-*N*′-(4-meth­oxy­benzo[*d*]thia­zol-2-yl)-3,6-di-p-tolyl-1,2,4-triazine-5-carboximidamide (**3**). There is six-membered intra­molecular resonance-assisted hydrogen bonding in the crystal structure of (**3**), which can be used in mol­ecular recognition (Mahmudov & Pombeiro, 2016 ▸).

## 2. Structural commentary

**Figure 1:**
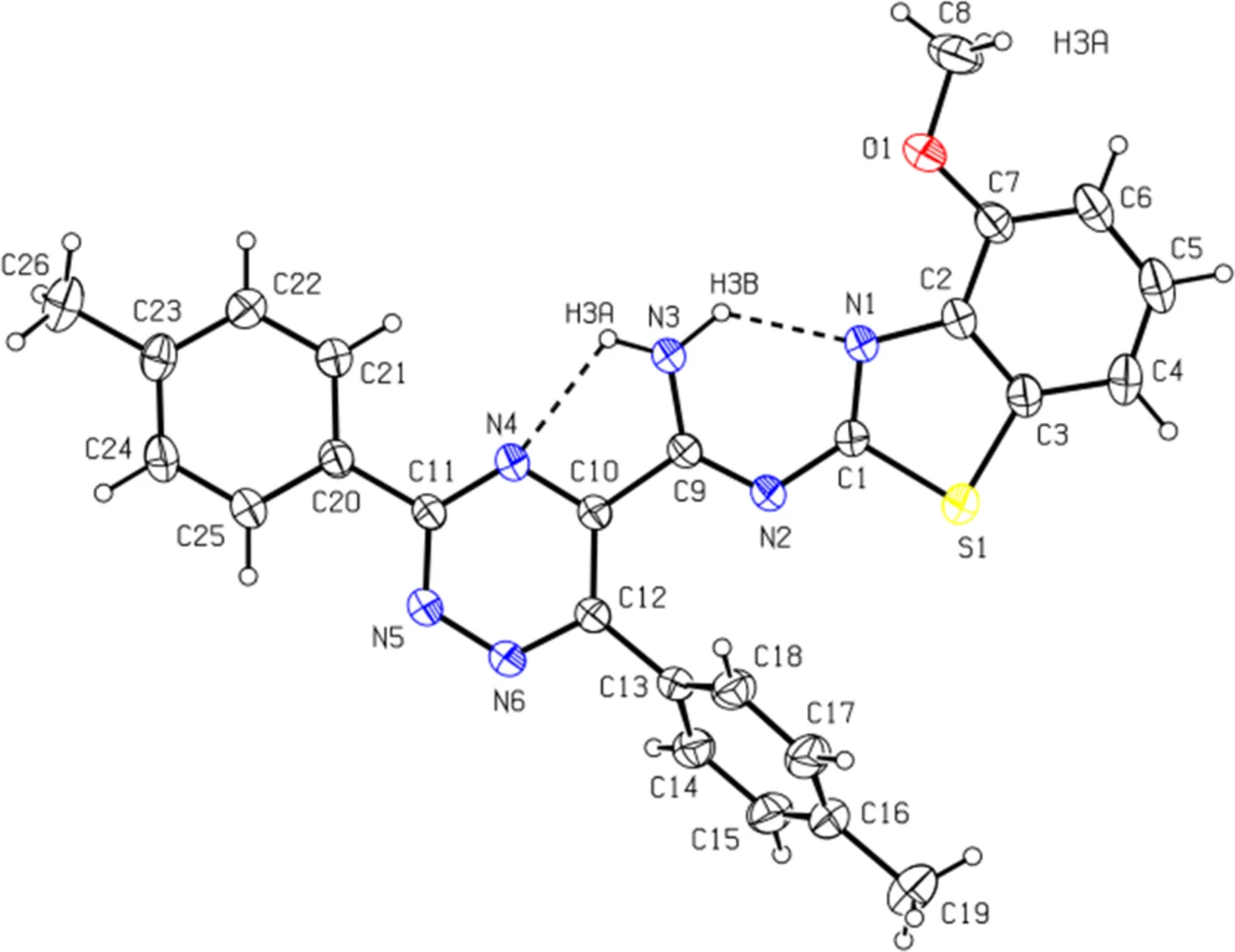
Mol­ecular structure of the title compound showing the atomic labelling. Displacement ellipsoids are drawn at the 30% probability level.

As shown Fig. 1 ▸, the conformation of the title mol­ecule is stabilized by two intra­molecular N—H⋯N hydrogen bonds, forming fused *S*(5) and *S*(6) rings (Table 1 ▸; Bernstein *et al.*, 1995 ▸). In a six-membered resonance-assisted hydrogen-bonding (RAHB) ring, the N3—H3*B*⋯N1 bond with [N⋯N distance = 2.663 (3) Å] falls in the N⋯N distance range [2.542–2.897 Å] observed for other supra­molecular synthons (Gurbanov *et al.*, 2020 ▸), and indicates a low delocalization within the heterodienic moiety. The N—H⋯N angle of 128 (2)° differs significantly from the average O—H⋯O angle (149°; Bertolasi *et al.*, 1993 ▸) of β-diketone enols involved in similar intra­molecular RAHB.

The 1,3-benzo­thia­zole ring system (S1/N1/C1–C7) is essentially planar with an r.m.s. deviation of 0.002 Å. The least-squares plane of this ring system forms dihedral angles of 8.18 (8), 64.96 (10), and 5.33 (9)°, respectively, with the planes of the 1,2,4-triazine ring (N4–N6/C10–C12) and the benzene rings (C13–C18 and C20–C25) of its two attached 4-methyl­phenyl groups. The benzene rings (C13–C18 and C20–C25) of the two 4-methyl­phenyl groups form angles of 68.68 (12)° with each other, while they make the angles of 57.10 (11) and 11.66 (10)°, respectively, with the 1,2,4-triazine ring plane (N4–N6/C10–C12). The bond lengths and angles in the title compound are in good agreement with those reported for related compounds (see *Database survey* section).

## 3. Supra­molecular features and Hirshfeld surface analysis

**Figure 2:**
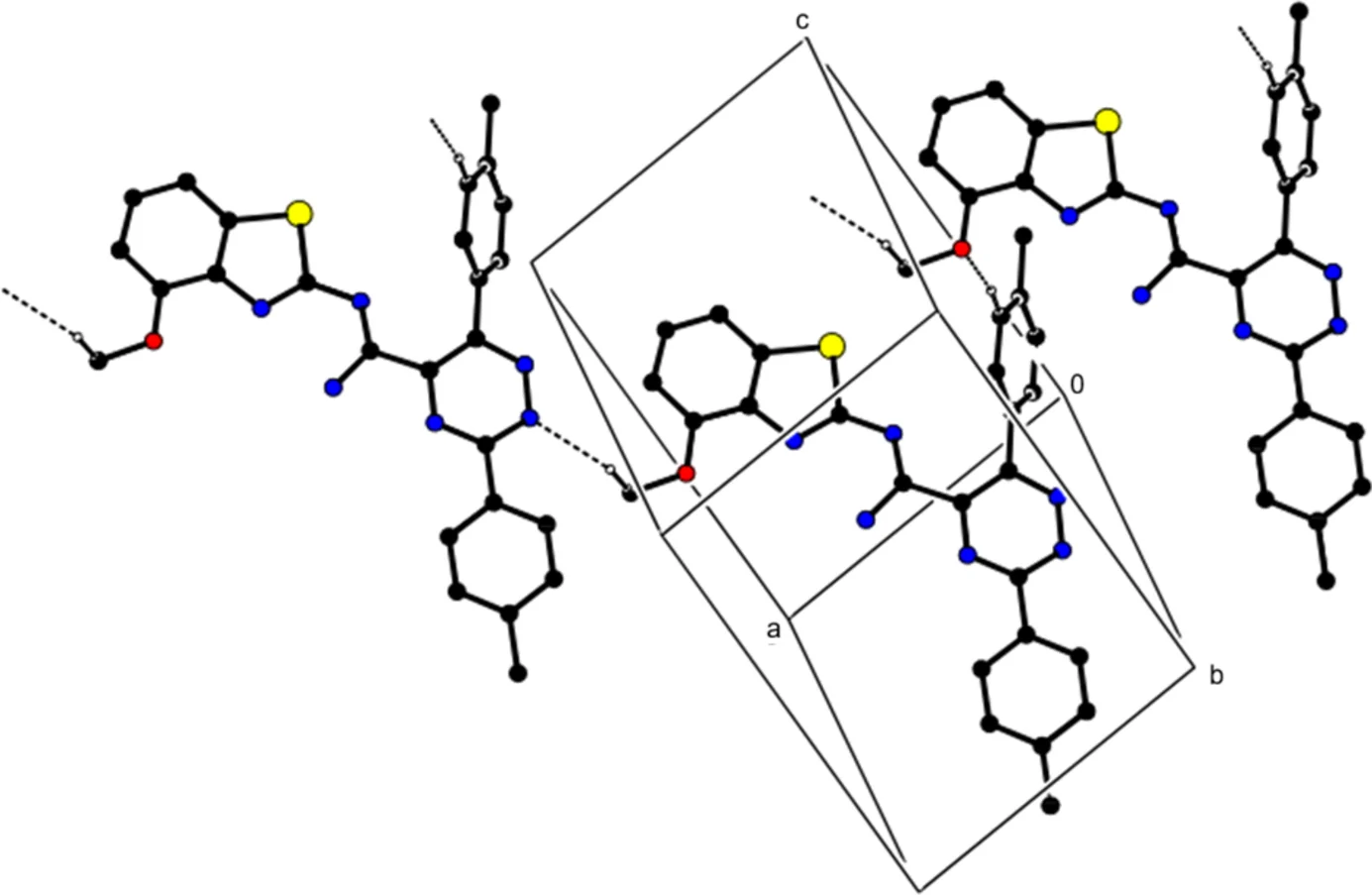
A partial view of the C—H⋯O and C—H⋯N bonds (dashed lines) in the unit cell. H atoms not involved in hydrogen bonding have been omitted.

**Figure 5:**
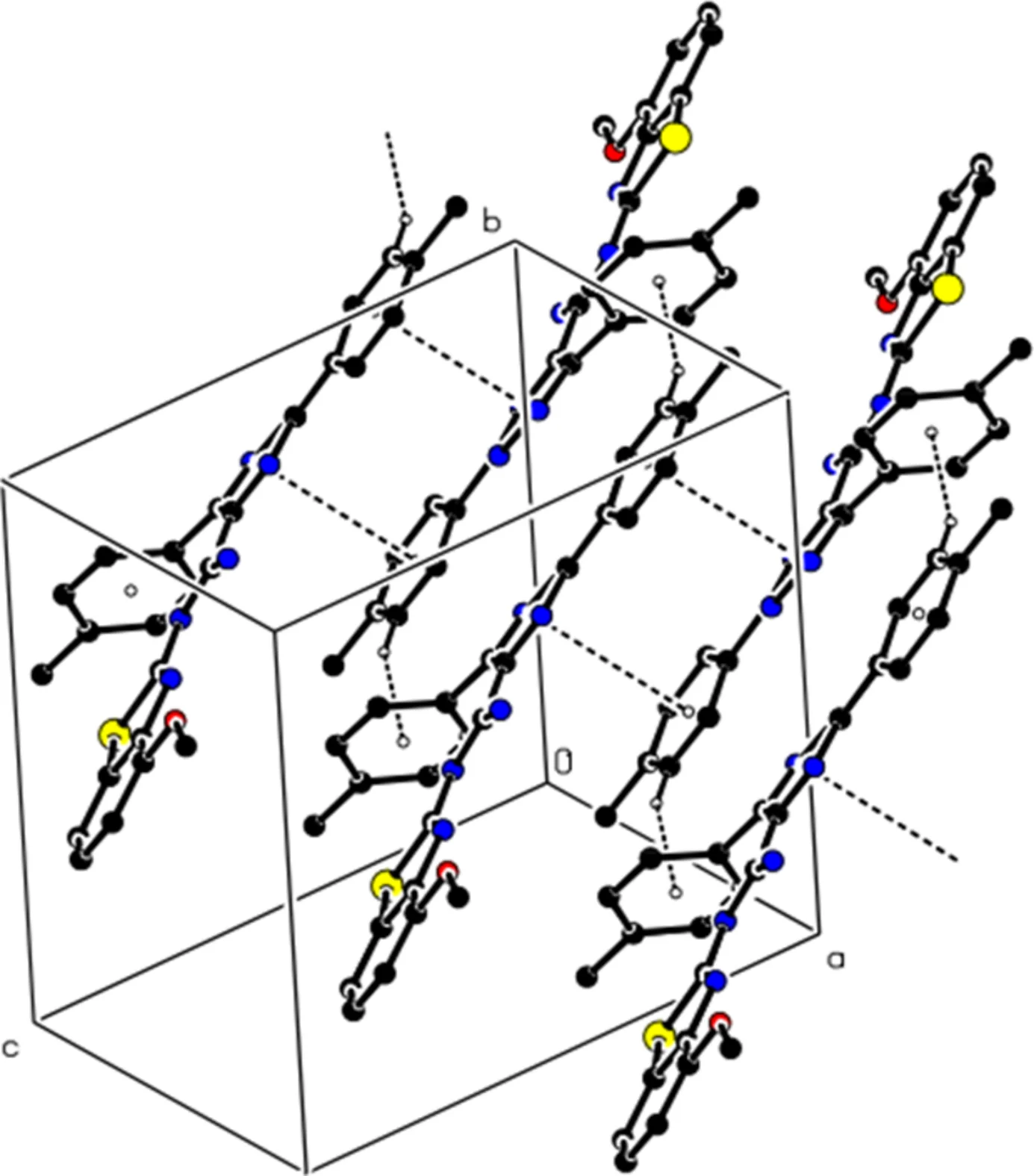
A view along the *a*-axis showing the C—H⋯π and π–π inter­actions (dashed lines).

In the crystal, the mol­ecules are linked by C—H⋯O and C—H⋯N hydrogen bonds, forming sheets parallel to the (010) plane (Figs. 2 ▸, 3 ▸ and 4 ▸; Table 1 ▸). The mol­ecules are further connected by C—H⋯π and π–π inter­actions [*Cg*2⋯*Cg*5^*a*^ = 3.7380 (12) Å, slippage = 1.201 Å; symmetry code (*a*) 1 − *x*, 1 − *y*, −*z*; where *Cg*2 and *Cg*5 are the centroids of the 1,2,4-triazine ring (N4–N6/C10–C12) and the benzene ring (C20–C25) of the 4-methyl­phenyl group, respectively], forming ribbons along the *a*-axis direction (Figs. 5 ▸ and 6 ▸; Table 1 ▸).

**Figure 7:**
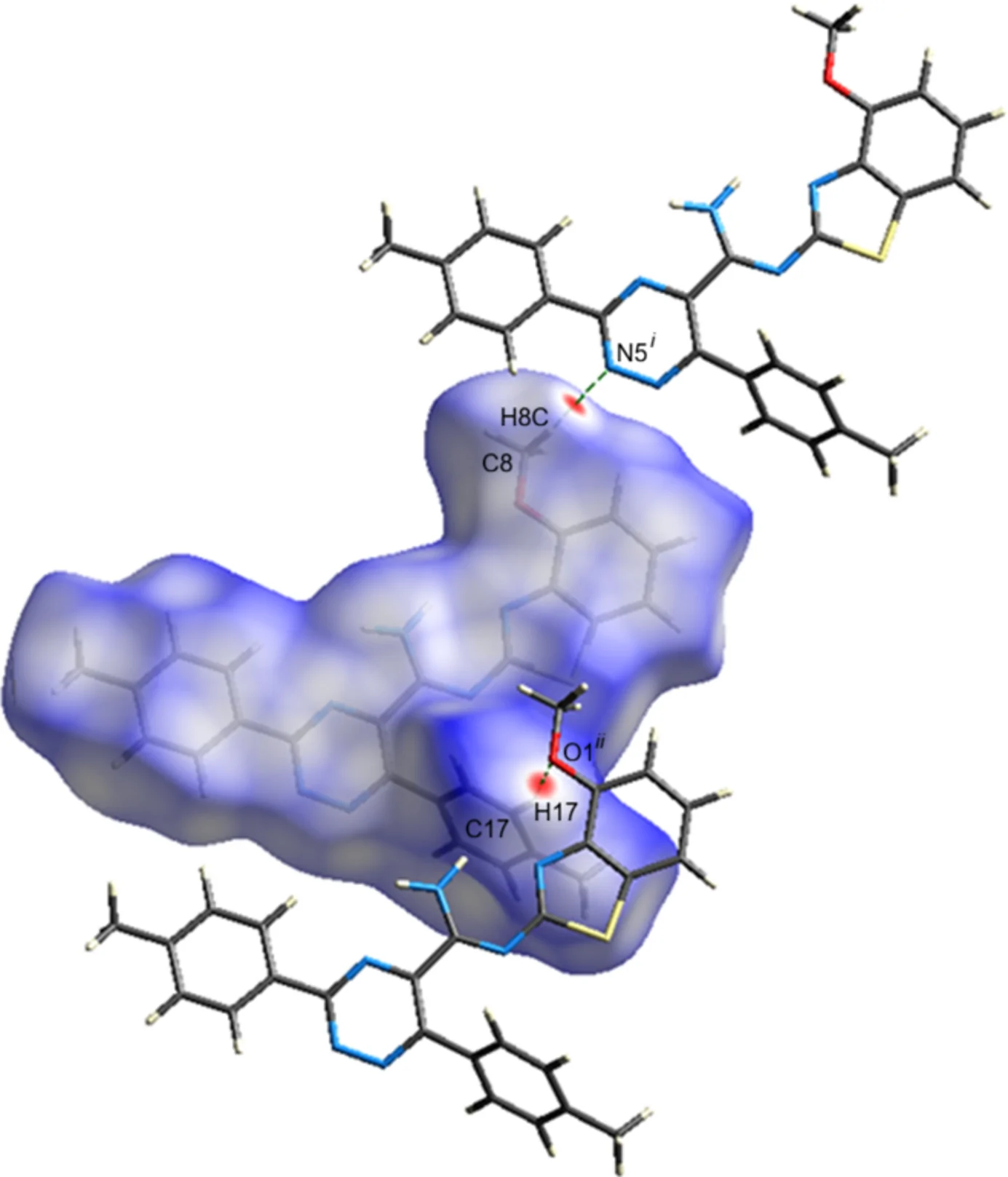
The Hirshfeld *d*_norm_ surface for the asymmetric unit with several neighboring mol­ecules. The C—H⋯O and C—H⋯N hydrogen bonds are depicted by red dashed lines.

**Figure 8:**
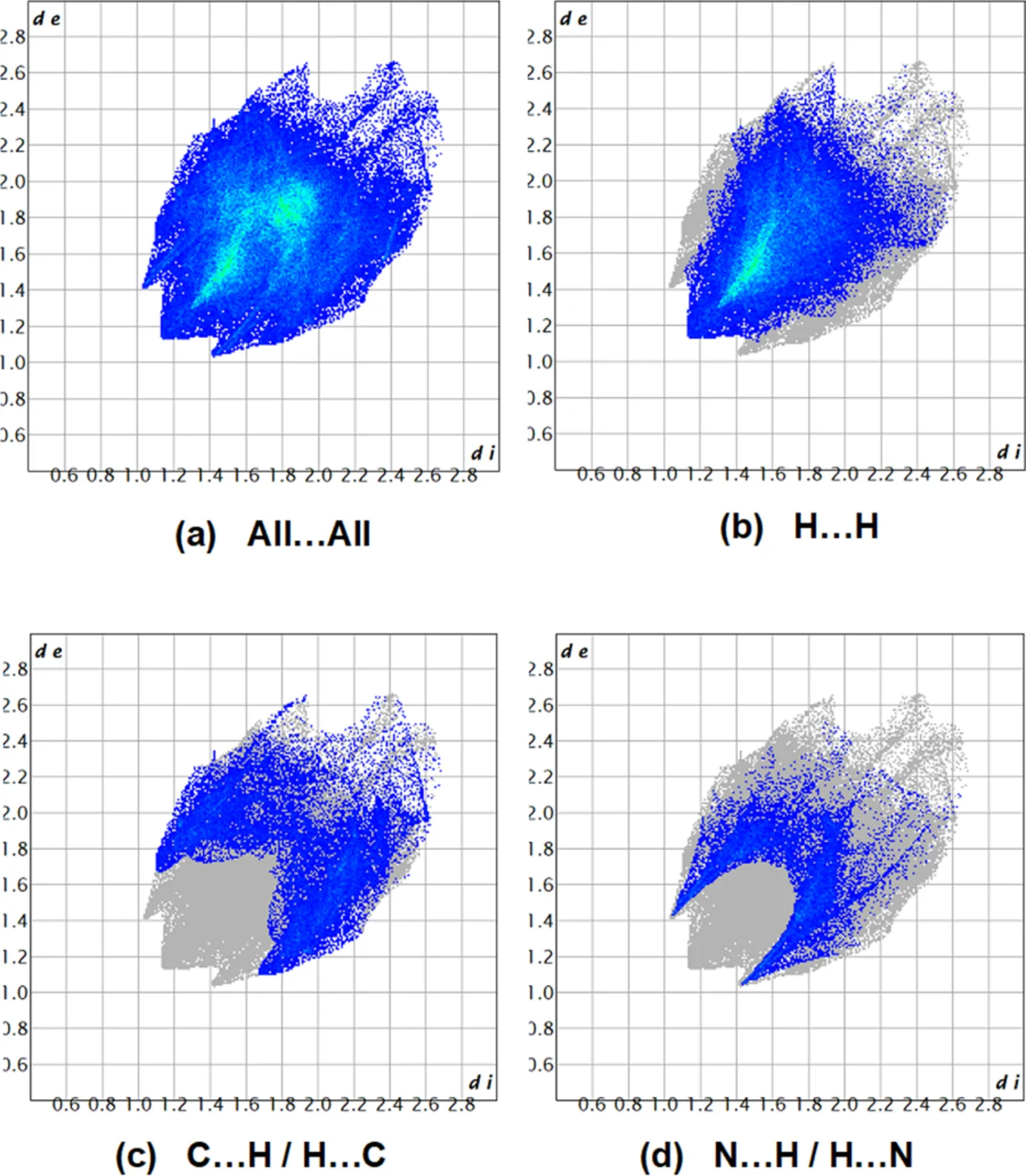
The two-dimensional fingerprint plots for the title compound showing (*a*) all inter­actions, and those delineated into (*b*) H⋯H, (*c*) C⋯H/H⋯C and (*d*) N⋯H / H⋯N inter­actions. The *d*_i_ and *d*_e_ values are the closest inter­nal and external distances (in Å) from given points on the Hirshfeld surface.

A Hirshfeld surface analysis was conducted in order to confirm the existence of hydrogen bonds and inter­molecular inter­actions (Table 2 ▸) in the crystal structure and evaluate the contributions from the various inter­molecular inter­actions. The three-dimensional Hirshfeld surface with a *d*_norm_ (normalized contact distance) plot and two-dimensional fingerprint plots were generated with *Crystal Explorer 17.5* (Spackman *et al.*, 2021 ▸). The Hirshfeld surface was plotted over the range −0.1153 (red) to +1.3440 (blue) a.u. (Fig. 7 ▸). The red spots on the surface indicate the sites of the C8—H8*C*⋯N5^i^ and C17—H17⋯O1^ii^ inter­actions. The two-dimensional fingerprint plots (Fig. 8 ▸) show that the most significant contacts are H⋯H (48.6%), C⋯H/H⋯C (18.8%) and N⋯H/H⋯N (12.0%) inter­actions. Smaller contributions are made by S⋯H/H⋯S (5.4%), C⋯C (5.3%), N⋯C/C⋯N (4.0%), S⋯C/C⋯S (1.1%), O⋯C/C⋯O (0.8%), N⋯N (0.7%), O⋯N/N⋯O (0.2%) and S⋯S (0.1%) inter­actions.

## 4. Database survey

A search of the Cambridge Structural Database (CSD, Version 6.00, update of April 2025; Groom *et al.*, 2016 ▸) for the *N′*-(1,3-benzo­thia­zol-2-yl)-1,2,4-triazine-5-carboximidamide unit revealed that the five compounds most closely related to the title compound are *N*-[(1,3-benzo­thia­zol-2-yl)carbamo­thio­yl]-4-bromo­benzamide (JOMGOL: Sow *et al.*, 2024 ▸), *N*′-(1,3-benzo­thia­zol-2-yl)benzene­sulfono­hydrazide (COCFOS: Baddeley *et al.*, 2019 ▸), *N*-(1,3-benzo­thia­zol-2-yl)acetamide (TIJPAF: Nayak *et al.*, 2013 ▸), 2-[(6-meth­oxy-1,3-benzo­thia­zol-2-yl)carbonoimido­yl]phenol (SUFFEG; Hijji *et al.*, 2015 ▸) and *N*-(4,6-di­methyl­pyrimidin-2-yl)-1,3-benzo­thia­zol-2-amine (YAJBUI: Mohamed *et al.*, 2011 ▸).

JOMGOL crystallizes in the monoclinic space group *P*2_1_/*n.* In the crystal, pairs of adjacent mol­ecules inter­act *via* inter­molecular hydrogen bonds of type C—H⋯N, C—H⋯S and N—H⋯S, resulting in mol­ecular layers parallel to the *ac* plane.

COCFOS crystallizes in the ortho­rhom­bic space group *Pbca*, with two mol­ecules (*A* and *B*) in the asymmetric unit. In the crystal, mol­ecules *A* and *B* form a supra­molecular dimer by pairwise N—H⋯N hydrogen bonding. The dimers are assembled into chains along the *a*-axis direction by N—H⋯O hydrogen bonds between *A* mol­ecules, whilst the mol­ecules are linked along [001] by linkages between centrosymmetrically related *B* mol­ecules. As a result, a wavy supermolecular layer is formed. C—H⋯O points of contact are visible as layers build along the *b* axis.

TIJPAF crystallizes in the monoclinic space group *P*2_1_/*c*, with two mol­ecules (*A* and *B*) in the asymmetric unit. In the crystal, pairs of N—H⋯N hydrogen bonds link the *A* and *B* mol­ecules into dimers, generating 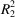
(8) loops. The dimers stack along [100].

SUFFEG crystallizes in the ortho­rhom­bic space group *Pna*2_1_, with two mol­ecules in the asymmetric unit. The two independent mol­ecules are linked by a pair of C—H⋯O hydrogen bonds, forming dimers with an 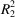
(20) ring motif. These dimers are further linked into sheets in the *ab* plane by weak inter­molecular C—H⋯N inter­actions.

YAJBUI crystallizes in the monoclinic space group *P*2_1_/*n.* The secondary amino N atom forms an inter­molecular N—H⋯N hydrogen bond to an N atom of the fused ring of an adjacent mol­ecule, generating a centrosymmetric cyclic hydrogen-bonded dimer with an 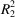
(8) motif.

## 5. Synthesis and crystallization

**Figure 9:**
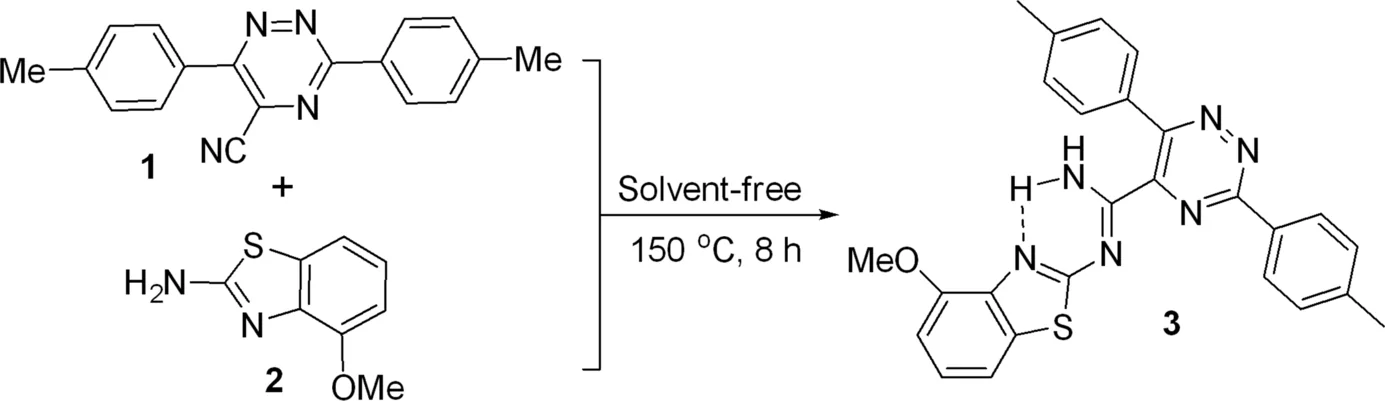
Synthesis of title compound.

The starting compound 5-cyano-1,2,4-triazine was prepared according to a literature procedure (Kozhevnikov *et al.*, 2002 ▸). 0.37 mmol of 3,6-di-*p*-tolyl-1,2,4-triazine-5-carbo­nitrile (**1**) and 0.41 mmol of 4-meth­oxy­benzo[*d*]thia­zol-2-amine (**2**) were added into a round-bottom flask and the resulting mixture was heated at 423 K for 8 h under inert atmosphere. The product was isolated by column chromatography (eluent AcOEt: CDCl_3_ = 1:9; *R*_f_ = 0.7 − 0.4) (Fig. 9 ▸). Analytical samples were obtained by recrystallization from ethanol solution. A single crystal of the title compound (**3**) was obtained by slow evaporation of its chloro­form solution.

Yield 69 mg (40%). ^1^H NMR (400 MHz, CDCl_3_): *δ* = 2.43 (*s*, 3H, CH_3_), 2.48 (*s*, 3H, CH_3_), 4.02 (*s*, 3H, OCH_3_), 6.85-6.91 (*m*, 1H, benzo­thia­zole), 7.19–7.28 (*m*, 3H, C_6_H_4_CH_3_+benzo­thia­zole), 7.28–7.33 (m, 1H, benzo­thia­zole), 7.36–7.41 (*m*, 2H, C_6_H_4_CH_3_), 7.53 (*br. s*, 1H, NH), 7.69–7.75 (*m*, 2H, C_6_H_4_CH_3_), 8.47–8.52 (*m*, 2H, C_6_H_4_CH_3_), 10.38 (*br. s*, 1H, NH). Mass-spectrum, *m*/*z* (*I*_rel_, %): *m*/*z*: 467.17 [*M*+H]^+^ (100). Found, %: C 66.91, H 4.73, N 18.02. C_26_H_22_N_6_OS. Calculated, %: C 66.93, H 4.75, N 18.01.

## 6. Refinement

Crystal data, data collection and structure refinement details are summarized in Table 3 ▸. The H atoms of the NH_2_ groups were found in difference-Fourier maps and were refined with *U*_iso_(H) = 1.2*U*_eq_(N). The C-bound H atoms were positioned geometrically (C—H = 0.93–0.96 Å) and refined using a riding model, with *U*_iso_(H) = 1.2 or 1.5*U*_eq_(C).

## Supplementary Material

Crystal structure: contains datablock(s) I. DOI: 10.1107/S2056989026007760/jp2030sup1.cif

Structure factors: contains datablock(s) I. DOI: 10.1107/S2056989026007760/jp2030Isup2.hkl

Supporting information file. DOI: 10.1107/S2056989026007760/jp2030Isup3.cml

CCDC reference: 2389378

Additional supporting information:  crystallographic information; 3D view; checkCIF report

## Figures and Tables

**Figure 3:**
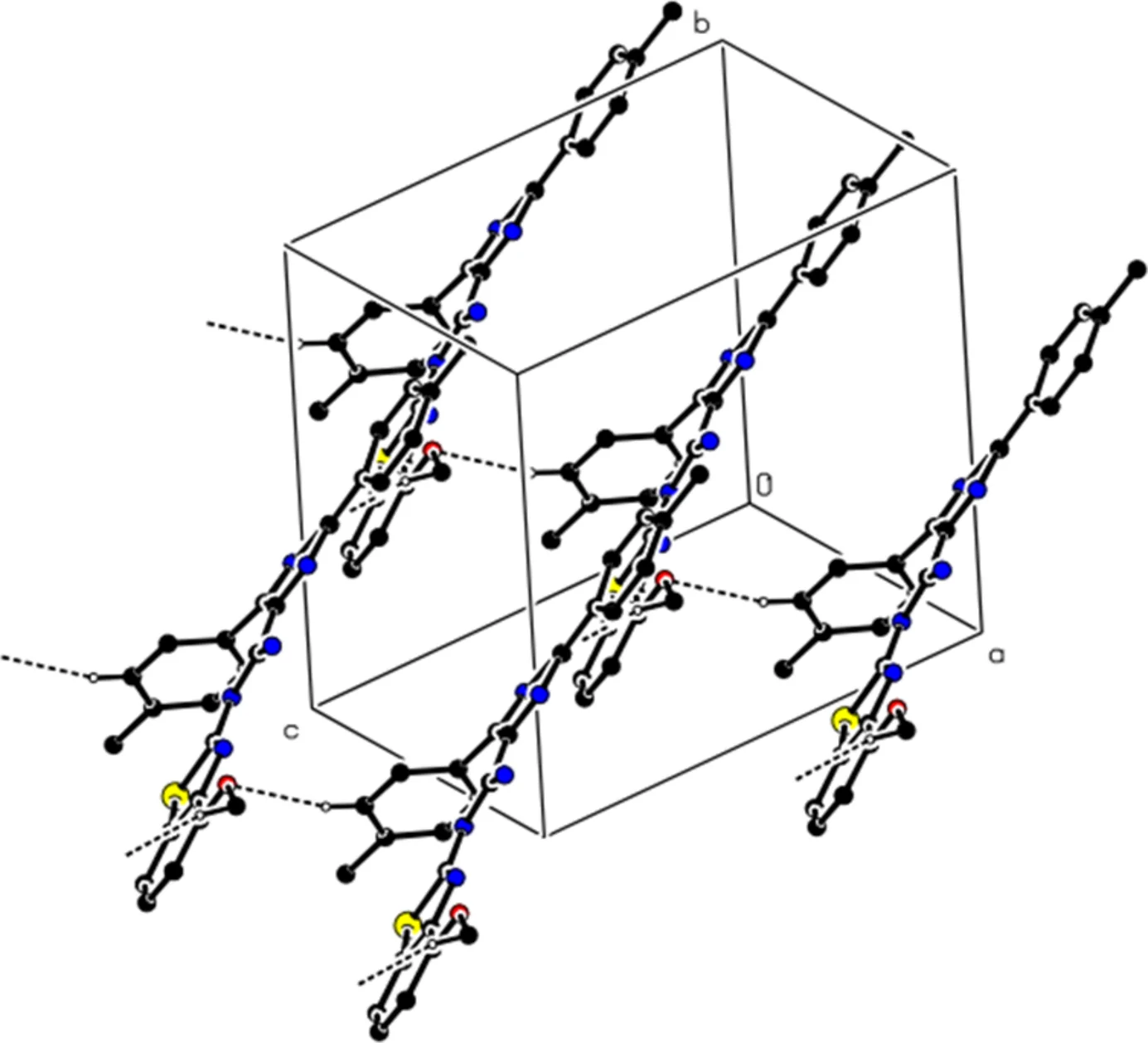
Crystal packing of the title compound viewed along the *a* axis showing the C—H⋯O and C—H⋯N bonds (dashed lines). H atoms not involved in hydrogen bonding have been omitted.

**Figure 4:**
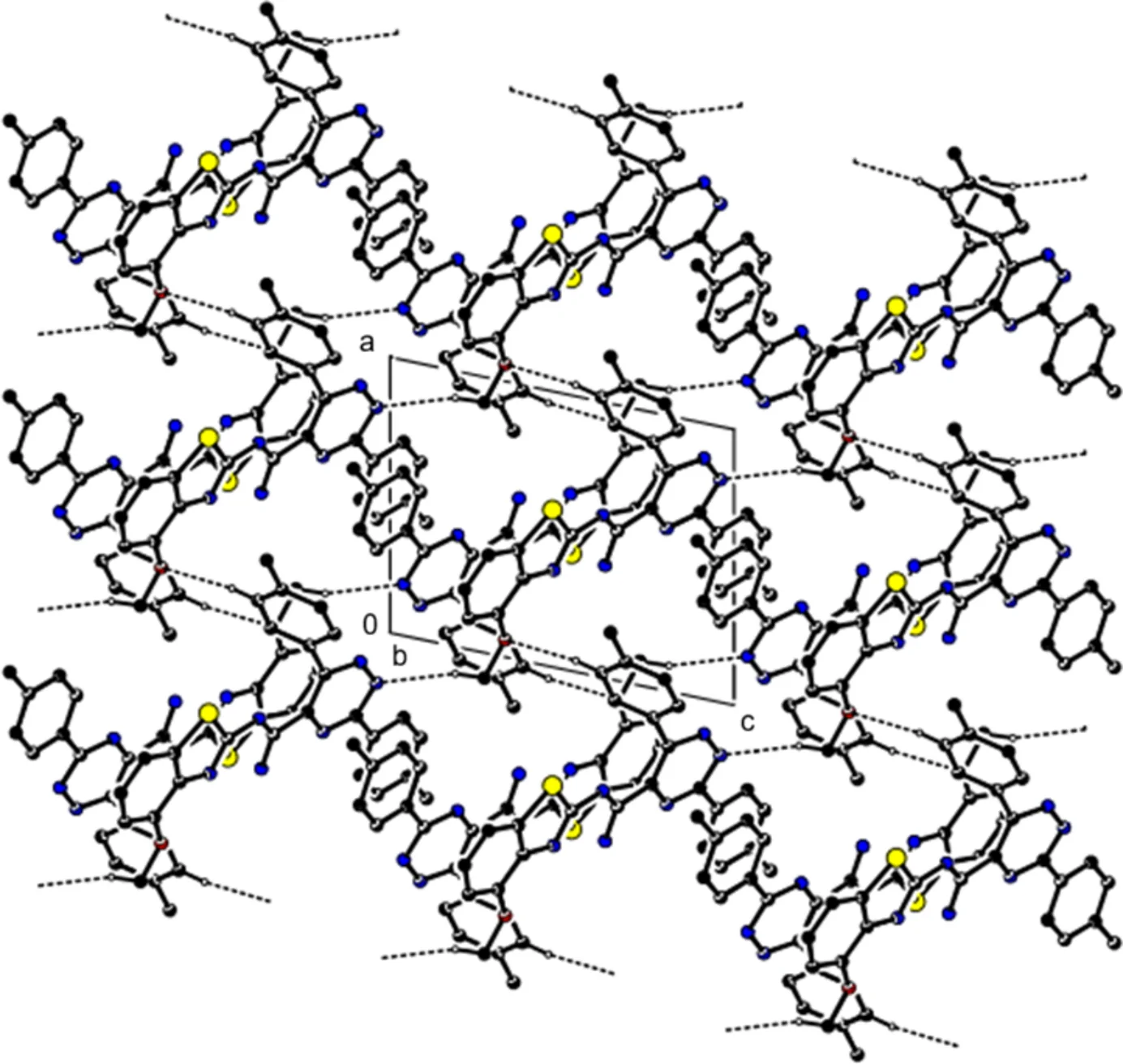
Crystal packing of the title compound viewed along the *b* axis showing the C—H⋯O and C—H⋯N bonds (dashed lines). H atoms not involved in hydrogen bonding have been omitted.

**Figure 6:**
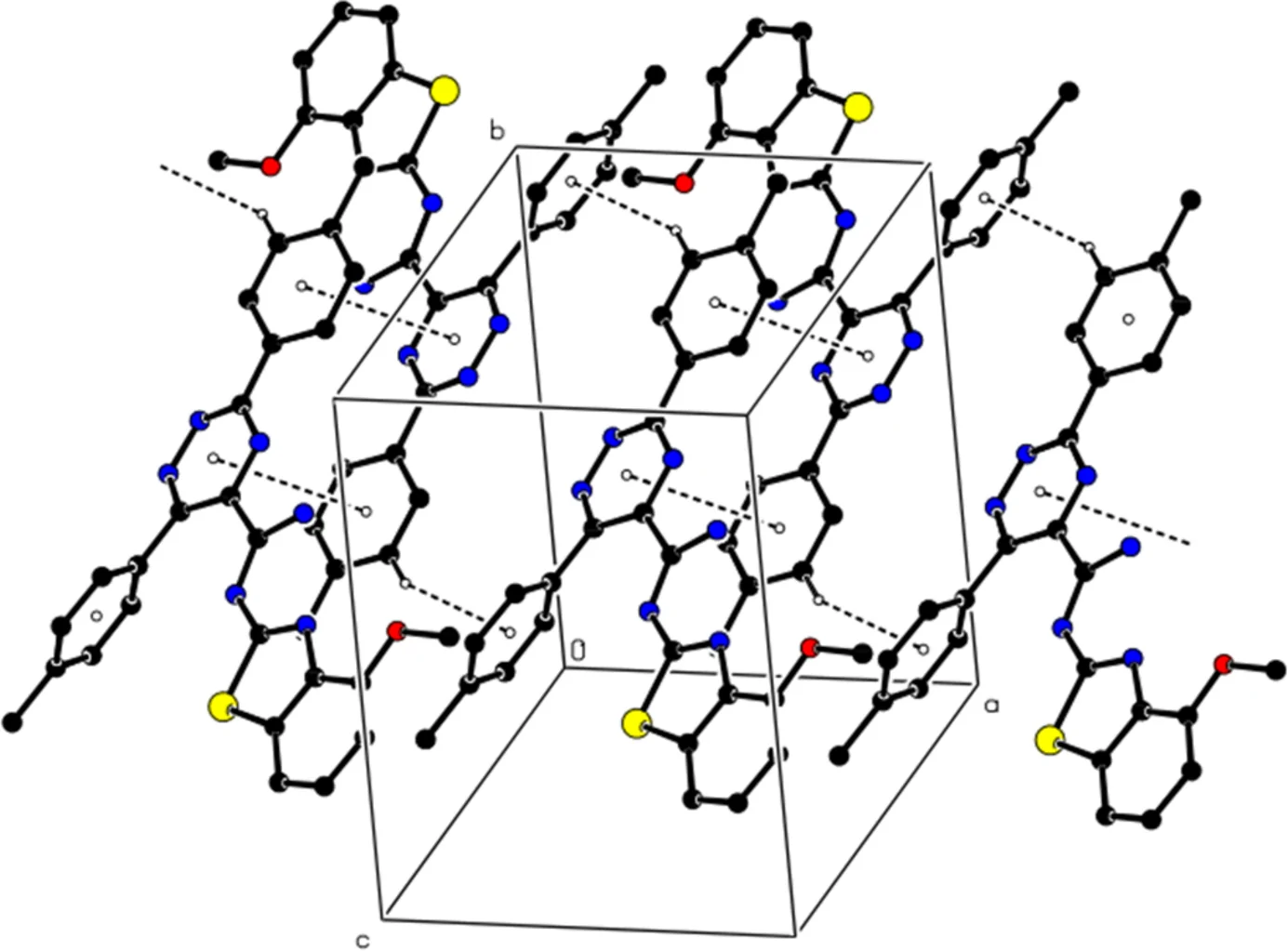
A view along the *c*-axis showing the C—H⋯π and π–π inter­actions (dashed lines).

**Table 1:** Hydrogen-bond geometry (Å, °) *Cg*4 is the centroid of the C13–C18 ring.

*D*—H⋯*A*	*D*—H	H⋯*A*	*D*⋯*A*	*D*—H⋯*A*
N3—H3*A*⋯N4	0.89 (2)	2.17 (2)	2.602 (3)	109 (2)
N3—H3*B*⋯N1	0.94 (2)	1.98 (2)	2.665 (3)	128 (2)
C8—H8*C*⋯N5^i^	0.96	2.60	3.532 (4)	163
C17—H17⋯O1^ii^	0.93	2.60	3.492 (3)	161
C24—H24⋯*Cg*4^iii^	0.93	2.96	3.796 (3)	151

Symmetry codes: (i) 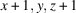; (ii) 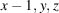; (iii) 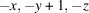.

**Table 2:** Summary of short inter­atomic contacts (Å)

Contact	Distance	Symmetry operation
C4⋯S1	3.67	1 − *x*, −*y*, 1 − *z*
H19*C*⋯·H19*C*	2.56	−*x*, −*y*, 1 − *z*
O1⋯H17	2.60	1 + *x*, *y*, *z*
H18⋯O1	2.87	1 − *x*, 1 − *y*, 1 − *z*
H26*C*⋯N2	2.68	1 − *x*, 1 − *y*, −*z*
H8*A*⋯H3*A*	2.53	2 − *x*, 1 − *y*, 1 − *z*
H25⋯H8*C*	2.43	−1 + *x*, *y*, −1 + *z*
H25⋯N6	2.81	−*x*, 1 − *y*, −*z*
H26*B*⋯H4	2.51	*x*, 1 + *y*, −1 + *z*
C26⋯C26	2.55	1 − *x*, 2 − *y*, −*z*

**Table 3:** Experimental details

Crystal data	
Chemical formula	C_26_H_22_N_6_OS
*M*_r_	466.55
Crystal system, space group	Triclinic, *P* 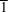
Temperature (K)	293
*a*, *b*, *c* (Å)	9.0055 (1), 11.5397 (1), 11.7583 (1)
α, β, γ (°)	103.401 (1), 100.311 (1), 95.215 (1)
*V* (Å^3^)	1158.22 (2)
*Z*	2
Radiation type	Mo *K*α
μ (mm^−1^)	0.17
Crystal size (mm)	0.41 × 0.28 × 0.13

Data collection	
Diffractometer	XtaLAB Synergy, Dualflex, HyPix
Absorption correction	Multi-scan (*CrysAlis PRO*; Rigaku OD, 2021 ▸)
*T*_min_, *T*_max_	0.745, 0.978
No. of measured, independent and observed [*I* > 2σ(*I*)] reflections	176835, 6298, 3926
*R*_int_	0.071
(sin θ/λ)_max_ (Å^−1^)	0.695

Refinement	
*R*[*F*^2^ > 2σ(*F*^2^)], *wR*(*F*^2^), *S*	0.050, 0.180, 1.13
No. of reflections	6298
No. of parameters	316
H-atom treatment	H atoms treated by a mixture of independent and constrained refinement
Δρ_max_, Δρ_min_ (e Å^−3^)	0.23, −0.33

Computer programs: *CrysAlis PRO* (Rigaku OD, 2021 ▸), *SHELXT* (Sheldrick, 2015*a* ▸), *SHELXL* (Sheldrick, 2015*b* ▸), *ORTEP-3 for Windows* (Farrugia, 2012 ▸) and *PLATON* (Spek, 2020 ▸).

## supplementary crystallographic information

### (Z)-N'-(4-Methoxybenzo[d]thiazol-2-yl)-3,6-di-p-tolyl-1,2,4-triazine-5-carboximidamide Crystal data

**Table:**

C_26_H_22_N_6_OS	*Z* = 2
*M**_r_* = 466.55	*F*(000) = 488
Triclinic, *P*1	*D*_x_ = 1.338 Mg m^−^^3^
*a* = 9.0055 (1) Å	Mo *K*α radiation, λ = 0.71073 Å
*b* = 11.5397 (1) Å	Cell parameters from 3380 reflections
*c* = 11.7583 (1) Å	θ = 1.8–25.2°
α = 103.401 (1)°	µ = 0.17 mm^−^^1^
β = 100.311 (1)°	*T* = 293 K
γ = 95.215 (1)°	Block, yellow
*V* = 1158.22 (2) Å^3^	0.41 × 0.28 × 0.13 mm

### (Z)-N'-(4-Methoxybenzo[d]thiazol-2-yl)-3,6-di-p-tolyl-1,2,4-triazine-5-carboximidamide Data collection

**Table:**

XtaLAB Synergy, Dualflex, HyPix diffractometer	6298 independent reflections
Radiation source: micro-focus sealed X-ray tube, PhotonJet (Mo) X-ray Source	3926 reflections with *I* > 2σ(*I*)
Mirror monochromator	*R*_int_ = 0.071
ω scans	θ_max_ = 29.6°, θ_min_ = 1.8°
Absorption correction: multi-scan (CrysAlisPro; Rigaku OD, 2021)	*h* = −12→12
*T*_min_ = 0.745, *T*_max_ = 0.978	*k* = −16→15
176835 measured reflections	*l* = −15→16

### (Z)-N'-(4-Methoxybenzo[d]thiazol-2-yl)-3,6-di-p-tolyl-1,2,4-triazine-5-carboximidamide Refinement

**Table:**

Refinement on *F*^2^	Primary atom site location: difference Fourier map
Least-squares matrix: full	Secondary atom site location: difference Fourier map
*R*[*F*^2^ > 2σ(*F*^2^)] = 0.050	Hydrogen site location: mixed
*wR*(*F*^2^) = 0.180	H atoms treated by a mixture of independent and constrained refinement
*S* = 1.13	*w* = 1/[σ^2^(*F*_o_^2^) + (0.0631*P*)^2^ + 0.5317*P*] where *P* = (*F*_o_^2^ + 2*F*_c_^2^)/3
6298 reflections	(Δ/σ)_max_ < 0.001
316 parameters	Δρ_max_ = 0.23 e Å^−^^3^
0 restraints	Δρ_min_ = −0.33 e Å^−^^3^

### (Z)-N'-(4-Methoxybenzo[d]thiazol-2-yl)-3,6-di-p-tolyl-1,2,4-triazine-5-carboximidamide Special details

**Table:**

Geometry. All esds (except the esd in the dihedral angle between two l.s. planes) are estimated using the full covariance matrix. The cell esds are taken into account individually in the estimation of esds in distances, angles and torsion angles; correlations between esds in cell parameters are only used when they are defined by crystal symmetry. An approximate (isotropic) treatment of cell esds is used for estimating esds involving l.s. planes.

### (Z)-N'-(4-Methoxybenzo[d]thiazol-2-yl)-3,6-di-p-tolyl-1,2,4-triazine-5-carboximidamide Fractional atomic coordinates and isotropic or equivalent isotropic displacement parameters (Å^2^)

**Table:**

	*x*	*y*	*z*	*U*_iso_*/*U*_eq_	
C1	0.5038 (2)	0.27699 (18)	0.47268 (18)	0.0465 (4)	
C2	0.6980 (2)	0.26819 (18)	0.61272 (17)	0.0466 (4)	
C3	0.5968 (3)	0.1778 (2)	0.6285 (2)	0.0550 (5)	
C4	0.6385 (3)	0.1141 (2)	0.7144 (2)	0.0705 (7)	
H4	0.570738	0.053160	0.724153	0.085*	
C5	0.7819 (3)	0.1445 (3)	0.7834 (2)	0.0748 (8)	
H5	0.811968	0.102707	0.840428	0.090*	
C6	0.8843 (3)	0.2356 (3)	0.7711 (2)	0.0668 (7)	
H6	0.980545	0.254748	0.820683	0.080*	
C7	0.8450 (2)	0.2984 (2)	0.68614 (19)	0.0530 (5)	
C8	1.0894 (3)	0.4134 (3)	0.7260 (3)	0.0911 (10)	
H8A	1.140292	0.479608	0.704830	0.137*	
H8B	1.137588	0.343166	0.703040	0.137*	
H8C	1.095260	0.432897	0.810727	0.137*	
C9	0.4539 (2)	0.39486 (18)	0.33990 (16)	0.0423 (4)	
C10	0.3467 (2)	0.42415 (17)	0.24019 (16)	0.0412 (4)	
C11	0.3239 (2)	0.53073 (18)	0.09882 (17)	0.0431 (4)	
C12	0.1965 (2)	0.36600 (19)	0.18987 (18)	0.0453 (4)	
C13	0.1038 (2)	0.28046 (19)	0.23459 (18)	0.0468 (5)	
C14	0.0420 (3)	0.1680 (2)	0.1617 (2)	0.0571 (5)	
H14	0.059479	0.146122	0.084447	0.068*	
C15	−0.0451 (3)	0.0881 (2)	0.2018 (3)	0.0662 (6)	
H15	−0.082782	0.012051	0.151942	0.079*	
C16	−0.0780 (3)	0.1179 (3)	0.3144 (3)	0.0667 (7)	
C17	−0.0205 (3)	0.2330 (3)	0.3851 (2)	0.0717 (7)	
H17	−0.044045	0.256997	0.460132	0.086*	
C18	0.0705 (3)	0.3125 (2)	0.3471 (2)	0.0610 (6)	
H18	0.109637	0.388064	0.397334	0.073*	
C19	−0.1738 (4)	0.0310 (3)	0.3591 (3)	0.1027 (11)	
H19A	−0.231054	−0.031130	0.292471	0.154*	
H19B	−0.242585	0.073161	0.401488	0.154*	
H19C	−0.108756	−0.004213	0.411681	0.154*	
C20	0.3869 (2)	0.62587 (18)	0.04898 (18)	0.0456 (4)	
C21	0.5380 (2)	0.67711 (19)	0.0873 (2)	0.0516 (5)	
H21	0.600772	0.652539	0.146502	0.062*	
C22	0.5965 (3)	0.7648 (2)	0.0380 (2)	0.0571 (5)	
H22	0.697977	0.798796	0.065626	0.069*	
C23	0.5077 (3)	0.80274 (19)	−0.0510 (2)	0.0545 (5)	
C24	0.3563 (3)	0.7517 (2)	−0.0881 (2)	0.0600 (6)	
H24	0.294026	0.776157	−0.147659	0.072*	
C25	0.2957 (3)	0.6656 (2)	−0.0388 (2)	0.0544 (5)	
H25	0.193187	0.633915	−0.064515	0.065*	
C26	0.5720 (4)	0.8962 (2)	−0.1063 (3)	0.0733 (7)	
H26A	0.652934	0.949887	−0.048577	0.110*	
H26B	0.493120	0.941006	−0.131536	0.110*	
H26C	0.610847	0.857396	−0.174114	0.110*	
N1	0.64364 (19)	0.32416 (15)	0.52443 (14)	0.0455 (4)	
N2	0.40818 (19)	0.30594 (16)	0.38132 (15)	0.0494 (4)	
N3	0.5885 (2)	0.46419 (19)	0.37481 (17)	0.0542 (5)	
H3A	0.598 (3)	0.527 (2)	0.344 (2)	0.065*	
H3B	0.664 (3)	0.445 (2)	0.431 (2)	0.065*	
N4	0.40762 (18)	0.50838 (15)	0.19569 (14)	0.0440 (4)	
N5	0.1895 (2)	0.46715 (18)	0.04002 (16)	0.0560 (5)	
N6	0.12501 (19)	0.38601 (18)	0.08723 (16)	0.0544 (5)	
O1	0.93428 (18)	0.39028 (16)	0.66585 (15)	0.0650 (4)	
S1	0.42638 (7)	0.16195 (6)	0.52779 (6)	0.0669 (2)	

### (Z)-N'-(4-Methoxybenzo[d]thiazol-2-yl)-3,6-di-p-tolyl-1,2,4-triazine-5-carboximidamide Atomic displacement parameters (Å^2^)

**Table:**

	*U*^11^	*U*^22^	*U*^33^	*U*^12^	*U*^13^	*U*^23^
C1	0.0459 (10)	0.0492 (11)	0.0447 (10)	0.0013 (8)	0.0035 (8)	0.0194 (9)
C2	0.0510 (11)	0.0504 (11)	0.0405 (10)	0.0120 (9)	0.0056 (8)	0.0165 (8)
C3	0.0611 (13)	0.0542 (12)	0.0529 (12)	0.0088 (10)	0.0064 (10)	0.0236 (10)
C4	0.0857 (18)	0.0673 (15)	0.0701 (16)	0.0128 (13)	0.0137 (14)	0.0415 (13)
C5	0.0890 (19)	0.0844 (19)	0.0641 (15)	0.0310 (16)	0.0092 (14)	0.0424 (14)
C6	0.0663 (15)	0.0840 (18)	0.0549 (13)	0.0268 (13)	0.0011 (11)	0.0295 (12)
C7	0.0496 (11)	0.0643 (13)	0.0455 (11)	0.0128 (10)	0.0051 (9)	0.0168 (10)
C8	0.0507 (14)	0.134 (3)	0.0775 (19)	−0.0033 (16)	−0.0105 (13)	0.0300 (19)
C9	0.0414 (10)	0.0470 (10)	0.0365 (9)	0.0014 (8)	0.0021 (7)	0.0128 (8)
C10	0.0391 (9)	0.0468 (10)	0.0378 (9)	0.0047 (8)	0.0047 (7)	0.0139 (8)
C11	0.0391 (9)	0.0503 (11)	0.0415 (10)	0.0079 (8)	0.0040 (7)	0.0176 (8)
C12	0.0391 (9)	0.0542 (11)	0.0433 (10)	0.0041 (8)	0.0044 (8)	0.0175 (9)
C13	0.0351 (9)	0.0580 (12)	0.0480 (11)	0.0033 (8)	0.0030 (8)	0.0203 (9)
C14	0.0505 (12)	0.0634 (14)	0.0570 (13)	0.0031 (10)	0.0116 (10)	0.0163 (11)
C15	0.0571 (14)	0.0591 (14)	0.0802 (17)	−0.0023 (11)	0.0105 (12)	0.0201 (12)
C16	0.0510 (13)	0.0774 (17)	0.0797 (17)	0.0009 (11)	0.0124 (12)	0.0397 (14)
C17	0.0647 (15)	0.095 (2)	0.0580 (14)	−0.0052 (14)	0.0196 (12)	0.0257 (14)
C18	0.0558 (13)	0.0708 (15)	0.0534 (13)	−0.0038 (11)	0.0129 (10)	0.0133 (11)
C19	0.093 (2)	0.108 (3)	0.114 (3)	−0.019 (2)	0.025 (2)	0.053 (2)
C20	0.0468 (10)	0.0481 (11)	0.0440 (10)	0.0097 (8)	0.0061 (8)	0.0167 (8)
C21	0.0478 (11)	0.0544 (12)	0.0556 (12)	0.0059 (9)	0.0056 (9)	0.0241 (10)
C22	0.0528 (12)	0.0550 (12)	0.0649 (14)	0.0020 (10)	0.0104 (10)	0.0212 (11)
C23	0.0694 (14)	0.0447 (11)	0.0591 (13)	0.0169 (10)	0.0240 (11)	0.0210 (10)
C24	0.0693 (15)	0.0609 (13)	0.0585 (13)	0.0197 (11)	0.0097 (11)	0.0312 (11)
C25	0.0497 (11)	0.0603 (13)	0.0559 (12)	0.0097 (10)	0.0020 (9)	0.0263 (10)
C26	0.097 (2)	0.0598 (14)	0.0801 (17)	0.0171 (13)	0.0397 (16)	0.0333 (13)
N1	0.0438 (9)	0.0511 (9)	0.0421 (9)	0.0035 (7)	0.0017 (7)	0.0195 (7)
N2	0.0452 (9)	0.0546 (10)	0.0455 (9)	−0.0020 (7)	−0.0034 (7)	0.0205 (8)
N3	0.0424 (9)	0.0663 (12)	0.0549 (11)	−0.0050 (8)	−0.0041 (8)	0.0331 (9)
N4	0.0444 (9)	0.0481 (9)	0.0401 (8)	0.0061 (7)	0.0034 (7)	0.0163 (7)
N5	0.0466 (10)	0.0694 (12)	0.0536 (10)	−0.0001 (8)	−0.0016 (8)	0.0311 (9)
N6	0.0412 (9)	0.0695 (12)	0.0534 (10)	−0.0004 (8)	−0.0007 (7)	0.0283 (9)
O1	0.0477 (9)	0.0818 (12)	0.0610 (10)	−0.0005 (8)	−0.0061 (7)	0.0260 (9)
S1	0.0618 (4)	0.0670 (4)	0.0726 (4)	−0.0104 (3)	−0.0026 (3)	0.0391 (3)

### (Z)-N'-(4-Methoxybenzo[d]thiazol-2-yl)-3,6-di-p-tolyl-1,2,4-triazine-5-carboximidamide Geometric parameters (Å, º)

**Table:**

C1—N1	1.304 (2)	C13—C18	1.383 (3)
C1—N2	1.377 (3)	C14—C15	1.376 (3)
C1—S1	1.750 (2)	C14—H14	0.9300
C2—N1	1.386 (2)	C15—C16	1.381 (4)
C2—C3	1.389 (3)	C15—H15	0.9300
C2—C7	1.410 (3)	C16—C17	1.390 (4)
C3—C4	1.400 (3)	C16—C19	1.508 (4)
C3—S1	1.729 (2)	C17—C18	1.377 (3)
C4—C5	1.363 (4)	C17—H17	0.9300
C4—H4	0.9300	C18—H18	0.9300
C5—C6	1.385 (4)	C19—H19A	0.9600
C5—H5	0.9300	C19—H19B	0.9600
C6—C7	1.379 (3)	C19—H19C	0.9600
C6—H6	0.9300	C20—C21	1.383 (3)
C7—O1	1.363 (3)	C20—C25	1.392 (3)
C8—O1	1.419 (3)	C21—C22	1.385 (3)
C8—H8A	0.9600	C21—H21	0.9300
C8—H8B	0.9600	C22—C23	1.379 (3)
C8—H8C	0.9600	C22—H22	0.9300
C9—N2	1.299 (3)	C23—C24	1.385 (3)
C9—N3	1.331 (2)	C23—C26	1.506 (3)
C9—C10	1.503 (3)	C24—C25	1.379 (3)
C10—N4	1.327 (2)	C24—H24	0.9300
C10—C12	1.412 (3)	C25—H25	0.9300
C11—N5	1.336 (3)	C26—H26A	0.9600
C11—N4	1.340 (2)	C26—H26B	0.9600
C11—C20	1.475 (3)	C26—H26C	0.9600
C12—N6	1.343 (3)	N3—H3A	0.89 (3)
C12—C13	1.482 (3)	N3—H3B	0.93 (3)
C13—C14	1.383 (3)	N5—N6	1.334 (2)
			
N1—C1—N2	128.93 (18)	C15—C16—C19	122.0 (3)
N1—C1—S1	115.58 (15)	C17—C16—C19	121.0 (3)
N2—C1—S1	115.49 (15)	C18—C17—C16	121.8 (2)
N1—C2—C3	116.05 (18)	C18—C17—H17	119.1
N1—C2—C7	124.46 (19)	C16—C17—H17	119.1
C3—C2—C7	119.48 (19)	C17—C18—C13	120.3 (2)
C2—C3—C4	121.5 (2)	C17—C18—H18	119.8
C2—C3—S1	109.14 (15)	C13—C18—H18	119.8
C4—C3—S1	129.4 (2)	C16—C19—H19A	109.5
C5—C4—C3	117.7 (2)	C16—C19—H19B	109.5
C5—C4—H4	121.1	H19A—C19—H19B	109.5
C3—C4—H4	121.1	C16—C19—H19C	109.5
C4—C5—C6	122.1 (2)	H19A—C19—H19C	109.5
C4—C5—H5	119.0	H19B—C19—H19C	109.5
C6—C5—H5	119.0	C21—C20—C25	118.3 (2)
C7—C6—C5	120.7 (2)	C21—C20—C11	121.12 (17)
C7—C6—H6	119.6	C25—C20—C11	120.58 (19)
C5—C6—H6	119.6	C20—C21—C22	120.5 (2)
O1—C7—C6	126.5 (2)	C20—C21—H21	119.8
O1—C7—C2	115.05 (18)	C22—C21—H21	119.8
C6—C7—C2	118.5 (2)	C23—C22—C21	121.6 (2)
O1—C8—H8A	109.5	C23—C22—H22	119.2
O1—C8—H8B	109.5	C21—C22—H22	119.2
H8A—C8—H8B	109.5	C22—C23—C24	117.5 (2)
O1—C8—H8C	109.5	C22—C23—C26	121.6 (2)
H8A—C8—H8C	109.5	C24—C23—C26	120.8 (2)
H8B—C8—H8C	109.5	C25—C24—C23	121.6 (2)
N2—C9—N3	127.22 (18)	C25—C24—H24	119.2
N2—C9—C10	118.00 (17)	C23—C24—H24	119.2
N3—C9—C10	114.77 (17)	C24—C25—C20	120.4 (2)
N4—C10—C12	120.41 (17)	C24—C25—H25	119.8
N4—C10—C9	113.95 (16)	C20—C25—H25	119.8
C12—C10—C9	125.54 (17)	C23—C26—H26A	109.5
N5—C11—N4	123.82 (18)	C23—C26—H26B	109.5
N5—C11—C20	117.49 (17)	H26A—C26—H26B	109.5
N4—C11—C20	118.57 (17)	C23—C26—H26C	109.5
N6—C12—C10	118.44 (18)	H26A—C26—H26C	109.5
N6—C12—C13	113.36 (17)	H26B—C26—H26C	109.5
C10—C12—C13	128.18 (17)	C1—N1—C2	110.04 (17)
C14—C13—C18	118.3 (2)	C9—N2—C1	119.35 (17)
C14—C13—C12	120.26 (19)	C9—N3—H3A	115.9 (17)
C18—C13—C12	121.4 (2)	C9—N3—H3B	118.7 (16)
C15—C14—C13	120.8 (2)	H3A—N3—H3B	125 (2)
C15—C14—H14	119.6	C10—N4—C11	117.28 (17)
C13—C14—H14	119.6	N6—N5—C11	118.75 (17)
C14—C15—C16	121.6 (2)	N5—N6—C12	120.45 (17)
C14—C15—H15	119.2	C7—O1—C8	117.1 (2)
C16—C15—H15	119.2	C3—S1—C1	89.20 (10)
C15—C16—C17	117.0 (2)		
			
N1—C2—C3—C4	179.6 (2)	N4—C11—C20—C21	11.0 (3)
C7—C2—C3—C4	−1.3 (4)	N5—C11—C20—C25	14.2 (3)
N1—C2—C3—S1	0.3 (2)	N4—C11—C20—C25	−169.59 (19)
C7—C2—C3—S1	179.36 (17)	C25—C20—C21—C22	−0.7 (3)
C2—C3—C4—C5	0.6 (4)	C11—C20—C21—C22	178.7 (2)
S1—C3—C4—C5	179.9 (2)	C20—C21—C22—C23	−0.8 (4)
C3—C4—C5—C6	0.6 (4)	C21—C22—C23—C24	1.3 (4)
C4—C5—C6—C7	−1.1 (4)	C21—C22—C23—C26	−178.8 (2)
C5—C6—C7—O1	179.3 (2)	C22—C23—C24—C25	−0.3 (4)
C5—C6—C7—C2	0.5 (4)	C26—C23—C24—C25	179.7 (2)
N1—C2—C7—O1	0.7 (3)	C23—C24—C25—C20	−1.1 (4)
C3—C2—C7—O1	−178.3 (2)	C21—C20—C25—C24	1.6 (3)
N1—C2—C7—C6	179.7 (2)	C11—C20—C25—C24	−177.8 (2)
C3—C2—C7—C6	0.7 (3)	N2—C1—N1—C2	179.9 (2)
N2—C9—C10—N4	−173.89 (18)	S1—C1—N1—C2	0.6 (2)
N3—C9—C10—N4	5.3 (3)	C3—C2—N1—C1	−0.5 (3)
N2—C9—C10—C12	2.6 (3)	C7—C2—N1—C1	−179.6 (2)
N3—C9—C10—C12	−178.2 (2)	N3—C9—N2—C1	1.0 (3)
N4—C10—C12—N6	8.8 (3)	C10—C9—N2—C1	−179.96 (18)
C9—C10—C12—N6	−167.53 (19)	N1—C1—N2—C9	−3.2 (4)
N4—C10—C12—C13	−172.7 (2)	S1—C1—N2—C9	176.18 (16)
C9—C10—C12—C13	11.0 (3)	C12—C10—N4—C11	−3.2 (3)
N6—C12—C13—C14	54.0 (3)	C9—C10—N4—C11	173.53 (17)
C10—C12—C13—C14	−124.6 (2)	N5—C11—N4—C10	−5.5 (3)
N6—C12—C13—C18	−122.7 (2)	C20—C11—N4—C10	178.55 (17)
C10—C12—C13—C18	58.7 (3)	N4—C11—N5—N6	8.4 (3)
C18—C13—C14—C15	−2.7 (3)	C20—C11—N5—N6	−175.61 (19)
C12—C13—C14—C15	−179.6 (2)	C11—N5—N6—C12	−2.2 (3)
C13—C14—C15—C16	2.0 (4)	C10—C12—N6—N5	−5.9 (3)
C14—C15—C16—C17	0.7 (4)	C13—C12—N6—N5	175.36 (19)
C14—C15—C16—C19	179.8 (3)	C6—C7—O1—C8	9.3 (4)
C15—C16—C17—C18	−2.6 (4)	C2—C7—O1—C8	−171.8 (2)
C19—C16—C17—C18	178.3 (3)	C2—C3—S1—C1	0.05 (17)
C16—C17—C18—C13	1.8 (4)	C4—C3—S1—C1	−179.3 (3)
C14—C13—C18—C17	0.9 (4)	N1—C1—S1—C3	−0.38 (18)
C12—C13—C18—C17	177.7 (2)	N2—C1—S1—C3	−179.81 (18)
N5—C11—C20—C21	−165.3 (2)		

### (Z)-N'-(4-Methoxybenzo[d]thiazol-2-yl)-3,6-di-p-tolyl-1,2,4-triazine-5-carboximidamide Hydrogen-bond geometry (Å, º)

*Cg*4 is the centroid of the C13–C18 ring.

**Table:**

*D*—H···*A*	*D*—H	H···*A*	*D*···*A*	*D*—H···*A*
N3—H3*A*···N4	0.89 (2)	2.17 (2)	2.602 (3)	109 (2)
N3—H3*B*···N1	0.94 (2)	1.98 (2)	2.665 (3)	128 (2)
C8—H8*C*···N5^i^	0.96	2.60	3.532 (4)	163
C17—H17···O1^ii^	0.93	2.60	3.492 (3)	161
C21—H21···N4	0.93	2.53	2.840 (3)	100
C24—H24···*Cg*4^iii^	0.93	2.96	3.796 (3)	151

Symmetry codes: (i) *x*+1, *y*, *z*+1; (ii) *x*−1, *y*, *z*; (iii) −*x*, −*y*+1, −*z*.
